# Pulmonary mucoepidermoid carcinoma: A clinicopathological study of 45 patients

**DOI:** 10.1111/1759-7714.14536

**Published:** 2022-06-23

**Authors:** Wang Shen, Tao Yang, Yaguang Fan, Xuebing Li, Cheng Ai, Xinyun Wang, Dan Wang, Xuexia Zhou

**Affiliations:** ^1^ Tianjin Lung Cancer Institute Tianjin Medical University General Hospital Tianjin China; ^2^ Department of Thoracic Surgery the First People's Hospital of Longquanyi District Chengdu Chengdu China; ^3^ Department of Cardiothoracic Surgery Bishan hospital of Chongqing Medical University Chongqing China; ^4^ Department of Pathology Tianjin Medical University General Hospital Tianjin China; ^5^ Tianjin Neurological Institute Tianjin Medical University General Hospital Tianjin China

**Keywords:** lung cancer, pathology, pulmonary mucoepidermoid carcinoma

## Abstract

Pulmonary mucoepidermoid carcinoma (PMEC) is uncommon. The purpose of this study was to evaluate the clinicopathological features, diagnostic criteria, treatment options, and prognostic factors relating to primary PMEC. Clinical data on 45 patients with primary PMEC were collected and analyzed retrospectively at Tianjin Medical University General Hospital and the First People’ Hospital of Longquanyi District Chengdu from January 2008 to December 2020. The 45 patients (25 males and 20 females) ranged in age from 22 to 72 years, with a median age of 49 and an average age of 47.7. All the patients underwent surgery, with 32 receiving only surgery and 13 receiving both surgery and postoperative chemotherapy. A total of 34 instances of low‐grade tumors and 11 cases of high‐grade tumors were discovered during postoperative pathological diagnosis. Forty‐five patients were followed for 13 to 78 months, and four died during this period. In all four instances, a lung infection unrelated to the tumor was determined to be the cause of death. The MAML2 gene translocation was detected in 40 of 45 patients, with 34 of them testing positive. Radical surgery with lymph node dissection is an efficient treatment for PMEC. The prognosis is poor for patients with advanced disease, a negative MAML2 gene translocation, lymph node metastases, and high‐grade tumors.

## Graphical abstract

The univariate analysis of the survival of patients with PMEC showed that TNM stage, histological grade, lymph node metastasis, and MAML2 gene translocation had significant influences on survival in patients with PMEC.

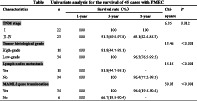

## INTRODUCTION

According to the World Health Organization's (WHO) lung tumor classification, primary salivary gland‐type tumors of the lung (PSGT) are a rare category of lung cancer with distinct clinical features that account for less than 1% of all primary lung tumors.^1^, ^2^ The most common subtype of PSGT is pulmonary mucoepidermoid carcinoma (PMEC), which is a malignant salivary gland‐type tumor composed of mucin‐secreting cells, squamoid cells, and intermediate‐type cells.^2^ The salivary glands, particularly the parotid gland, are commonly affected. However, it is rare in the lungs, accounting for just 0.1%–0.2% of all primary pulmonary malignant tumors.^3^ Due to the rarity of the condition, little is known regarding its demographics, treatment, prognosis, and survival. This study evaluated the results of 45 patients who underwent surgery for PMEC.

## METHODS

### Patients

From January 2008 to December 2020, 45 instances of patients with primary mucoepidermoid carcinoma of the lung were gathered from the clinical surgical archives of the First People’ Hospital of Longquanyi District Chengdu and Tianjin Medical University General Hospital. All patients underwent complete resection with lobectomy, bronchial sleeve resection, or resection and reconstruction of the tracheal carina and main bronchus, and 13 of the patients had chemotherapy after surgery. They were pathologically identified with primary MEC of the lung after surgery, according to the 2020 WHO classification and diagnostic criteria for thoracic tumors, with no previous history of salivary gland tumors, and no tumor on salivary gland imaging examination. The eighth edition of the international TNM staging method was used to stage all of the tumors after surgery. Throughout the follow‐up, all patients or family members were contacted by phone and asked whether they had survived, if they had died, the reason and date of death, and if there were any indicators of recurrence or complications.

### Statistical analysis

Descriptive statistics were used to show the distribution of personal and clinical characteristics of patients. One, 3 and 5‐year overall survival rates by personal and clinical characteristics were calculated with the Kaplan–Meier method, and compared statistically using the log‐rank test. Statistical analysis was performed using IBM SPSS Statistics, version 23.0 (IBM Corporation). Statistical significance was defined as a two‐sided *p*‐value of less than 0.05.

## RESULTS

The 45 patients included 25 males and 20 females, ranging in age from 22 to 72 years. The age of male patients ranged from 33 to 72 years, with a median of 52 years and an average of 53.3 years. Female patients were aged 22 to 54 years, with a median of 42 years and an average of 40.8 years. There were 20 non‐smokers and 25 smokers among the 45 patients. Patients presented with coughing in 18 cases, hemoptysis in nine cases, dyspnea in seven cases, chest pain in three cases, chest tightness in three cases, throat discomfort in one case, and no obvious symptoms in four cases. In terms of tumor location, 41 cases were central, and four cases were peripheral. According to the eighth edition of the worldwide TNM staging system, there were 22 cases classified as stage I, 19 cases classified as stage II, three cases classified as stage III, and one case classified as stage IV. (Table 1).

**TABLE 1:** The clinical characteristics of 45 patients with pulmonary mucoepidermoid carcinoma (PMEC)

Characteristics	*n*
Gender	
Male	25
Female	20
Smoking history	
Yes	25
No	20
Range	
Central	41
Peripheral	4
Tumor location	
Right main bronchus	5
Left main bronchus	3
Right upper lobe	10
Right middle lobe	1
Right inferior lobe	6
Left upper lobe	12
Left inferior lobe	8
Initial symptoms	
Cough	18
Hemoptysis	9
Dyspnea	7
Chest pain	3
Chest tightness	3
Throat discomfort	1
Asymptomatic	4
TNM stage	
Stage I	22
Stage II	19
Stage III	3
Stage IV	1
Histological grade	
High‐grade	11
Low‐grade	34
Lymph nodes metastasis	
Yes	11
No	34
Treatment	
Radical surgery	32
Radical surgery + postoperative chemotherapy	13
MAML2 gene translocation	
Yes	34
No	6

Gross pathological examination revealed that the specimens ranged in size from 1.5 × 1.0 × 0.8 cm to 5.5 × 3.2 × 1.8 cm, the section was gray white with a medium texture, some mucus was visible, a small cystic cavity was occasionally visible, and the boundary was still clear, but some boundaries were irregular.

**FIGURE 1:**
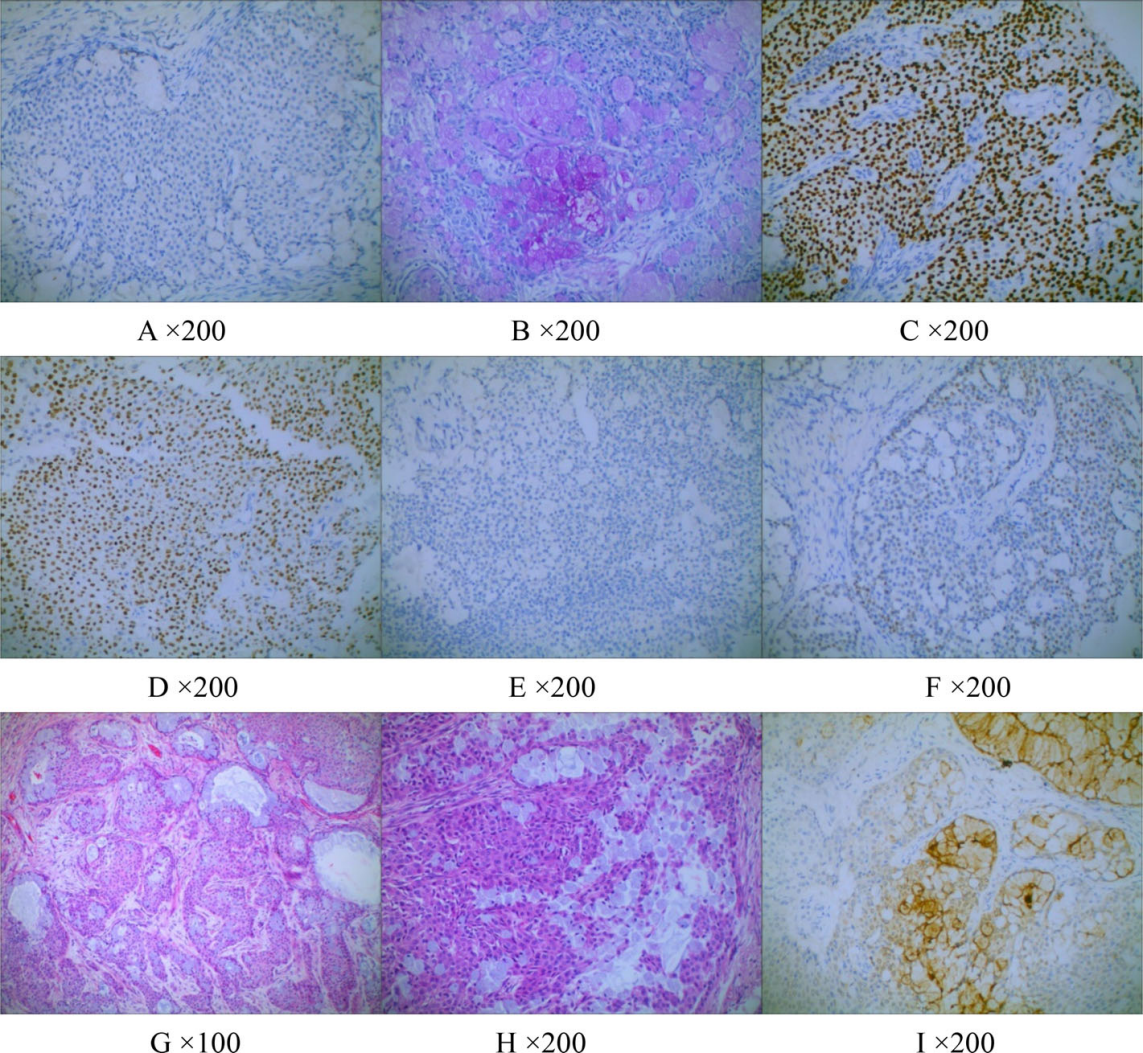
(a) TTF‐1 expression was negative, (b) AB/PAS was positive in the cytoplasm of mucus cells, (c,d) p63 and p40 were positive in the nuclei of intermediate and epidermoid cells, (e) napsin A expression was negative, (f) Ki‐67 expression was positive in tumor nuclei, (g) mucus cells were arranged in a glandular tube‐like way, (h) MEC contained three types of cells: mucus, epidermoid and intermediate cells, and (i) CK7 was positive in mucus cells

Microscopic observation indicated that the cancer tissue was composed of three different cell types (mucus, epidermoid, and intermediate cells). A total of 45 cases were categorized into two groups based on their proportions and degree of atypia: (1) Low‐grade: 34 occurrences (34/45), consisting of a tangled collection of glands, tubules, cysts, and solid areas, the majority of which were glandular tubes with mucus‐secreting cells and goblet cells. Intermediate and epidermal‐like cells were scattered inside a solid nest or sheet structure. Intercellular bridges and keratosis could be found in epidermal‐like cells. These two cell types have fewer components, exhibit no obvious atypia, and nuclear division and necrosis are infrequent. (2) High‐grade: In 11 (11/45) of the cases, the cancer cells were mostly intermediate and epidermoid cells, with just a few mucus cells. At times, the mucus‐filled capsule could be seen, as well as some scattered and single mucus cells. Atypia was prominent in intermediate and epidermoid cells, and nuclear division and necrosis were readily visible. Immunohistochemical staining was positive for CK7, p63, and p40, but negative for TTF‐1 and napsin A. Cytohistochemical staining revealed AB/PAS was positive **(**Figure 1 **)**. The Ki67 index varied from 2% to 30%, with an average of 23.5% for high‐grade patients and 3.2% for low‐grade patients.

Of the 45 cases, 11 cases of lymph node metastasis were found, whereas 34 cases were judged to be negative. In 40 of 45 cases, MAML2 gene translocation was detected, with 34 cases being negative and six cases being positive **(**Table 1 **)**.

All patients had complete mass resection with lobectomy (37 cases), bronchial sleeve resection (3 cases), or resection and reconstruction of the tracheal carina and main bronchus (5 cases). According to tumor histological grade (high‐grade), TNM stage (III–IV stage) and lymph node metastasis (positive), 13 of the patients also had chemotherapy after surgery. The chemotherapy regimen included docetaxel + cisplatin (5 cases), gemcitabine + cisplatin (5 cases), and pemetrexed + cisplatin (4 cases).

A total of 45 patients were followed‐up over a period of 13 to 78 months. Four patients died of pulmonary infection unrelated to recurrence at 13, 16, 22, and 28 months after surgery, respectively.

In our study, univariate analysis showed 3‐year survival rates of 100% in the patients with TNM stage I versus 91.3% in patients with TNM stage II–IV, and 5‐year survival rates of 100% in patients with TNM stage I versus 69.1% in the patients with TNM stage II–IV. In addition, 3‐year survival rates of 100% in the patients with low‐grade versus 81.8% in patients with high‐grade tumors, and 3‐year survival rates of 81.8% in patients with lymph node metastasis versus 100% in patients with no lymph node metastasis. Three‐year survival rates were 100% in the patients with MAML2 gene translocation versus 66.7% in the patients with no MAML2 gene translocation (Table 2).

**TABLE 2:** Univariate analysis of survival rates of 45 cases with pulmonary mucoepidermoid carcinoma (PMEC)

Characteristics	n	Survival rate (%)			Chi‐square	*p*
		1‐year	3‐year	5‐year		
Gender					0.20	0.653
Male	25	100	96.0 (74.8–99.4)	85.7 (61.4–95.3)		
Female	20	100	94.7 (68.1–99.2)	82.8 (55.4–94.2)		
Age (year)					0.18	0.668
≥45	27	100	94.7 (68.1–99.2)	88.0 (59.4–96.9)		
<45	18	100	96.0 (74.8–99.4)	81.4 (56.7–92.8)		
Smoking history					0.20	0.658
Yes	25	100	90.0 (65.6–97.4)	84.7 (59.7–94.8)		
No	20	100	100	84.9 (59.5–94.9)		
TNM stage					6.35	0.012
I	22	100	100	100		
II–IV	23	100	91.3 (69.5–97.8)	69.1 (42.4–85.3)		
Tumor histological grade					13.46	<0.001
High‐grade	11	100	81.8 (44.7–95.1)	–		
Low‐grade	34	100	100	96.3 (76.5–99.5)		
Lymph node metastasis					15.15	<0.001
Yes	11	100	81.8 (44.7–95.1)	–		
No	34	100	100	96.4 (77.2–99.5)		
Treatment					2.223	0.455
Radical surgery	32	93.8	87.5	18.8		
Radical surgery + postoperative chemotherapy	13	92.3	69.2	0.00		
MAML2 gene translocation					30.03	<0.001
Yes	34	100	100	96.4 (19.5–90.4)		
No	6	100	66.7 (19.5–90.4)	–		

## DISCUSSION

Salivary gland‐type neoplasms of the lung have been recognized since the 1950s. In the early 1960s, researchers discovered that these tumors had the potential of invasive development and metastasis but were less aggressive than the more common bronchogenic carcinomas.^4^, ^5^ Due to their histological similarities to salivary tumors originating in the head and neck, these tumors are now believed to originate from the salivary glands of the tracheobronchial tree. Mucoepidermoid carcinoma of the lungs is a subclass of salivary gland‐type neoplasms that accounts for 0.1–0.2 percent of all pulmonary malignancies. The majority of PMECs arise in the proximal bronchi.

The univariate analysis of the survival of patients with PMEC showed that gender, age, smoking history, and therapy had no significant influence on survival in patients with PMEC (*p*>0.05), but TNM stage, histological grade, lymph node metastasis, and MAML2 gene translocation did (*p*<0.05, <0.001, <0.001, <0.001). (Table 2).

MECs in the lungs tend to occur people of different ages. The average age in our series was 47.7 years. According to the 2020 WHO classification of thoracic tumors and certain series, there is a female predominance and a broad age range,^2^ but in our study, we found no indication of significant gender and age disparities. Moreover, we discovered no apparent relationship between this condition and smoking.

Due to its origin and location, PMEC is commonly endobronchial and therefore patients often have signs and symptoms of airway obstruction or irritation, such as cough, shortness of breath, or dyspnea. However, some individuals are asymptomatic.

Additionally, some individuals may be misdiagnosed with bronchial asthma due to the presence of symptoms that are similar to those of bronchial asthma, and more attention should be paid to this in certain patients.

On high‐resolution computer tomography (HRCT) images, lesions of PMEC were often round, oval, or lobulated nodules or masses with a well‐defined border. The densities of tumors were comparable to those of muscle. The majority of enhanced scans demonstrated visible enhancement, while a few had very little change.^6^ Our cases demonstrated that calcification may occur in certain tumors, most notably some pathological low‐grade malignancies. According to Johnson et al.,^6^ this phenomenon may be associated with the inability of the tumor to absorb mucus generated by mucus cells, which results in calcium salt accumulation. Due to the increased number of mucus cells in low‐grade PMEC, calcification may be more frequent. In addition, PMEC should be differentiated from primary bronchus leiomyoma and adenoid cystic carcinoma (ACC). Although bronchial leiomyoma presents as a polypoid nodule, it does not develop in strips along the inner wall of the airway. ACC infiltrates the airway wall and thickens the tracheal wall annularly.

Due to the rarity of PMEC and the lack of specificity in clinical and imaging features, diagnosis is mostly reliant on sophisticated histological characteristics. PMEC should be distinguished from adenosquamous carcinoma, particularly in tiny biopsy specimens obtained through fiberoptic bronchoscopy or lung puncture. In order to diagnose MEC, three types of cells must be present: mucoepidermoid, epidermoid, and intermediate cells. (Figure 1) Furthermore, adjunct immunohistochemistry and cytochemistry staining techniques, as well as MAML2 gene translocation analysis, may be performed to aid the diagnosis. TTF‐1 and napsin A are frequently positive in adenosquamous cell carcinoma but not in PMEC, thus assisting differentiation. Huo et al.^7^ discovered that Ki‐67 variation varied from 2% to 80%, with an average of 9.7%. Ki‐67 accounted for 22.4% in high‐grade PMEC, while Ki‐67 accounted for 4.1% in low‐grade PMEC. The Ki‐67 index in our cases ranged from 2% to 30%, with an average of 23.5% for high‐grade patients and 3.2% for low‐grade patients.

According to previous studies, complete surgical resection and lymph node dissection have been considered crucial for long‐term survival in patients with PMEC.^8^, ^9^, ^10^ Patients with positive lymph node metastases, positive tumor margins, or patients with a high‐grade type should undergo postoperative chemotherapy.^11^ However, the state of adjuvant radiation after surgery is uncertain.^7^ The majority of studies believe surgical adjuvant radiation or chemotherapy to be unnecessary for low‐grade pulmonary MEC.^11^ Our research showed that the TNM stage, histological grade, lymph node metastasis, and MAML2 gene translocation all had a significant effect on the prognosis of PMEC patients.

We acknowledge the limitations of this study. Some potential prognostic factors may have been missed due to the limited sample size. For example, we were unable to compare surgical treatment or surgery plus postoperative chemotherapy with chemotherapy or radiotherapy alone.

In conclusion, PMEC is uncommon, lacks identifiable symptoms and CT signals, is often identified postoperatively, and should be differentiated from adenosquamous carcinoma. Complete surgical resection with lymph node dissection is an effective treatment option. Patients with advanced disease, those with a negative MAML2 gene translocation, with lymph node metastases, and high‐grade malignancies have a poor prognosis.

## CONFLICT OF INTEREST

No authors report any conflict of interest in this work.
